# A Disclaimer

**Published:** 1887-03

**Authors:** 


### A DISCLAIMER.

We have always understood an "official organ," of any party or body, to be a paper which, authorized, or unauthorized, paid or unpaid, publishes the official papers and documents of that body, and advocates its course and principles.

The editor of this JOURNAL, occupying, through accident of death of Dr. Burt, the position of Secretary of the Texas State Medical Association, (and from his office official documents are issued), felt that the claim could be made, without offense, that the JOURNAL, which always publishes any official document of the Association—all news, or matters relating to the Association, gratuitously; and which advocates, first, last, and always, ORGANIZATION, into a grand State Association of the entire regular profession of the State, could properly be called its official organ, without objection, as, under the circumstances, it virtually is, *de facto* if not *de jure;* but we have never claimed to have received any authority from the Association to so consider the JOURNAL.

A few years ago, Dr. Wilkinson's journal, the *Record*, was the official organ, and published all the proceedings. That method was abolished, and the Transactions have been published in book form ever since. Meantime, this JOURNAL has been established, and for the purpose of advocating the cause of the Association; and its editor has become, as stated, the Secretary. The Association has about doubled its membership, and there are many things to be published, besides the proceedings,—official papers, circulars and addresses, action of committees, etc., and medical news of interest to the members; hence the aspect is changed, and there is necessity for some exponent of the Association, other than its scientific work and proceedings as recorded in the yearly Transactions.

Moreover, no claim has been made to exclusive privilege in the matter of publishing these things, holding, as we have said, that any journal which chose to publish such official papers, championing the cause and principles of the body, could be considered in the light of an official paper.

Now, since we learn that objection has been made by certain members, and our claim disputed, we will haul down the sign, and no longer claim to be the organ of anybody, but INDEPENDENT in all things.

We will not cease, however, to advocate ORGANIZATION, as the correct policy through which, alone, we can hope ever to acquire sufficient influence, and power to procure the much needed legislation in the interest of public health; nor will we cease to labor in the cause of the advancement and elevation of medical science in the Lone Star State;—that was the mission for which the JOURNAL was created,—its *raison d'etre*, and so long as we can raise a voice, or wield a pen, it will be in the interest of legitimate medicine, and the cause of the Texas State Medical Association!

